# Combined Neutrase–Alcalase Protein Hydrolysates from Hazelnut Meal, a Potential Functional Food Ingredient

**DOI:** 10.1021/acsomega.2c07157

**Published:** 2022-12-23

**Authors:** Fatma Duygu Ceylan, Nabil Adrar, Deniz Günal-Köroğlu, Büşra Gültekin Subaşı, Esra Capanoglu

**Affiliations:** † Department of Food Engineering, Faculty of Chemical and Metallurgical Engineering, Istanbul Technical University, 34469Istanbul, Turkey; ‡ Biology and Biological Engineering, Division of Food and Nutrition Science, Chalmers University of Technology, SE-412 96Gothenburg, Sweden

## Abstract

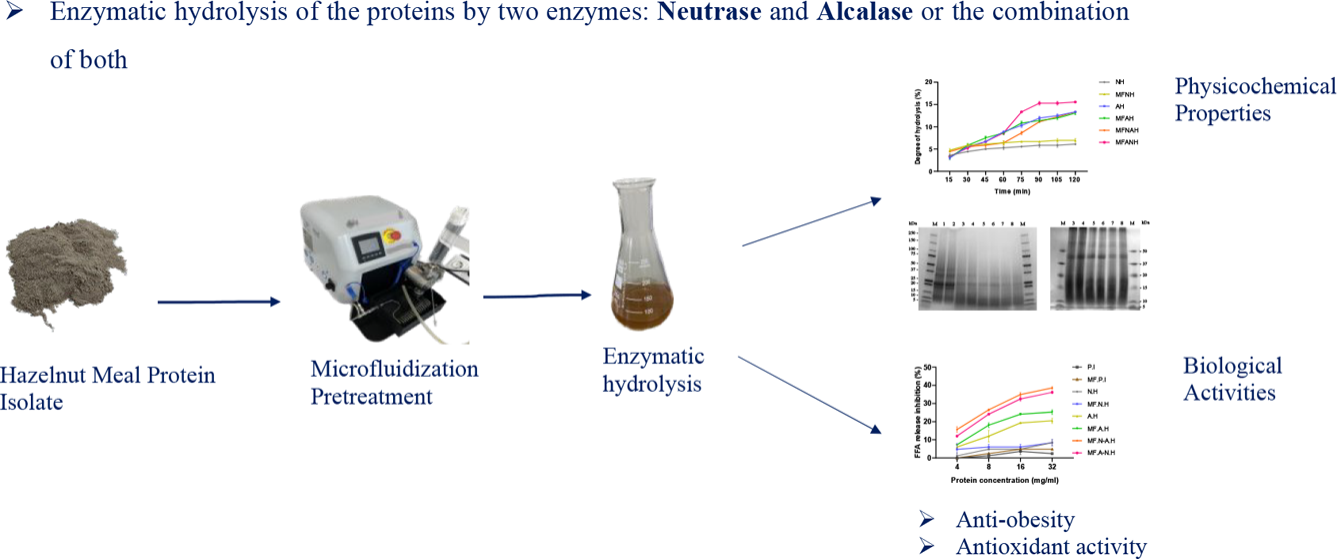

Consumers’ interest in functional foods has significantly increased in the past few years. Hazelnut meal, the main valuable byproduct of the hazelnut oil industry, is a rich source of proteins and bioactive peptides and thus has great potential to become a valuable functional ingredient. In this study, hazelnut protein hydrolysates obtained by a single or combined hydrolysis by Alcalase and Neutrase were mainly characterized for their physicochemical properties (SDS-PAGE, particle size distribution, Fourier-transform infrared (FTIR) spectroscopy, molecular weight distribution, etc.) and potential antiobesity effect (Free fatty acid (FFA) release inhibition), antioxidant activity (DPPH and ABTS methods), and emulsifying properties. The impact of a microfluidization pretreatment was also investigated. The combination of Alcalase with Neutrase permitted the highest degree of hydrolysis (DH; 15.57 ± 0.0%) of hazelnut protein isolate, which resulted in hydrolysates with the highest amount of low-molecular-weight peptides, as indicated by size exclusion chromatography (SEC) and SDS-PAGE. There was a positive correlation between the DH and the inhibition of FFA release by pancreatic lipase (PL), with a significant positive effect of microfluidization when followed by Alcalase hydrolysis. Microfluidization enhanced the emulsifying activity index (EAI) of protein isolates and hydrolysates. Low hydrolysis by Neutrase had the best effect on the EAI (84.32 ± 1.43 (NH) and 88.04 ± 2.22 m^2^/g (MFNH)), while a negative correlation between the emulsifying stability index (ESI) and the DH was observed. Again, the combined Alcalase–Neutrase hydrolysates displayed the highest radical scavenging activities (96.63 ± 1.06% DPPH and 98.31 ± 0.46% ABTS). FTIR results showed that the application of microfluidization caused the unfolding of the protein structure. The individual or combined application of the Alcalase and Neutrase enzymes caused a switch from the β-sheet organization of the proteins to α-helix structures. In conclusion, hazelnut meal may be a good source of bioactive and functional peptides. The control of its enzymatic hydrolysis, together with an appropriate pretreatment such as microfluidization, may be crucial to achieve the best suitable activity.

## 1 Introduction

Hazelnuts, widely cultivated in Turkey, constitute one of the most produced and consumed nuts worldwide.^1^ A big part of hazelnut production is processed industrially, mainly to produce hazelnut oil, leaving large quantities of hazelnut meal.^2^ Hazelnut meal is very rich in proteins (38–54%)^3,4^ and thus may be a cheap source of proteins that can be either introduced in food formulations or used as a base to produce bioactive peptides after enzymatic hydrolysis.

Plant-based proteins often have compact quaternary and tertiary structures, which may make them resistant to proteolysis.^5^ Microfluidization is a novel physical modification technique for processing food macromolecules with benefits such as continuous operation, low-temperature treatment, reduced nutrient component loss, and quick processing times.^6^ In addition, microfluidization as a pretreatment may improve the functional properties of proteins and their enzymatic hydrolysis, with the protein structure becoming more loosened, exposing core groups hidden inside the folded structure.^5−8^

Obesity and overweight are both major risk factors for several noncommunicable chronic diseases, including cardiovascular diseases, which are the leading causes of death worldwide, diabetes, and musculoskeletal disorders and are even associated with some cancers, including liver, prostate, endometrial, breast, ovarian, gallbladder, colon, and kidney.^9^ According to the last statistics from the World Health Organization, 1.9 billion adults worldwide were overweight in 2016, with 650 million being obese.^10^ Pancreatic lipase (PL) is the primary enzyme responsible for the hydrolysis of dietary fats (50–70%), which facilitates their absorption.^11^ This is why inhibiting PL is a good strategy to limit intestinal absorption of lipids and thus contribute to weight loss or prevent weight gain. In addition, plant-derived PL inhibitors are known resources that can lead to the selection of future drug candidates and thus enrich the current limited availability of antiobesity drugs.^12,13^ In recent years, bioactive peptides from edible plants have emerged as very promising and safe candidates as PL inhibitors which may also be introduced in functional food formulations with antiobesity potential.^14−17^

This study aimed to valorize hazelnut meal and explore the potential antiobesity and antioxidant activities of its protein hydrolysates. In addition, the effect of the hydrolysis strategy (single or sequential hydrolysis) using Alcalase and Neutrase, as well as the application of microfluidization pretreatment on these activities and the functional properties of the protein isolates and hydrolysates, was investigated.

## 2 Materials and Methods

### 2.1 Enzymes and Chemicals

Alcalase 2.4 L FG and Neutrase 0.8 L were obtained from Novozymes (Bagsværd, Denmark) and type II lipase from porcine pancreas (100–650 units/mg protein using olive oil and 30 min incubation) and bile salts (B8756-100G), sodium dodecyl sulfate (SDS), DPPH (2,2-diphenyl-1-picrylhydrazyl), and ABTS (2,2′-azino-di(3-ethylbenzthiazoline sulfonic acid)) were purchased from Sigma–Aldrich. (St. Louis, Missouri, USA). Other reagents were purchased from Merck (Darmstadt, Germany), and only analytical-grade reagents were used for analysis.

### 2.2 Hazelnut Meal Characterization

Hazelnut meal from *Corylus avellana* L. species was kindly donated by a local company in Ordu, Turkey. The meal was obtained as a byproduct of cold press oil extraction followed by hexane defatting. A proximate analysis of the meal was characterized according to standard methods.^18^ The total carbohydrate content was calculated by subtracting total protein, lipid, ash, and moisture percentages from 100. Protein, fat, ash, and total calculated carbohydrates of the hazelnut meal were 35.52 ± 1.72%, 3.64 ± 0.33%, 7.84 ± 0.09%, and 41.65 ± 2.20% (dry basis), respectively. Moisture represented 11.37 ± 0.02% of the meal.

### 2.3 Protein Extraction from Hazelnut Meal

Crude hazelnut proteins were isolated from hazelnut meal by the conventional alkali extraction–isoelectric precipitation method as described in the literature with slight modifications.^19,20^ The meal was first suspended in distilled water (1:12 meal:water), and the pH of the slurry was adjusted to 12 with 2 N NaOH. The mixture was then stirred for 1 h at room temperature. After centrifugation at 14480*g* for 10 min (25 °C), the supernatant was collected and the pellets were subjected to two further extractions. After filtration of the supernatants (Whatman grade-4 filter paper to remove eventual low-density particles), proteins were precipitated upon pH adjustment to the isoelectric point (4.5) with 2 N HCl and further centrifugation for 5 min at the same centrifuge force. For easier solubilization of the dried protein isolates, the precipitate was redissolved in water, and the pH was adjusted to 7 before lyophilization. The protein contents of the hazelnut meal and protein isolate were estimated using the Kjeldahl method (nitrogen content × 5.18; AOAC:^18^ method 950.48). The protein recovery from hazelnut meal was calculated as the ratio of the proteins extracted from 100 g of dry meal to the protein content of the meal, multiplied by 100. Crude proteins isolated were 22.75 ± 1.18% of the dry meal. Considering a purity of 62.12 ± 0.22% and the actual protein content in the meal (35.52 ± 1.72%), protein recovery was estimated to be 39.87 ± 3.99%. The purity of the isolates was considered in the calculation of the degree of hydrolysis (DH) of the proteins.

### 2.4 Preparation of Hazelnut Protein Hydrolysates

Hazelnut protein isolates (PI) were hydrolyzed by Alcalase and Neutrase separately or sequentially combined in both orders. A microfluidization pretreatment was applied to tentatively improve the hydrolysis and functionality of the proteins. PI was solubilized in distilled water at a concentration of 3% with the pH adjusted to 8. Microfluidized samples were passed two times through the microfluidizer (LM10, Microfluidics, Westwood, MA, USA) at 120 MPa.^21^ The enzymatic hydrolysis conditions were set with respect to the manufacturer’s recommendations. Briefly, hydrolysis by Neutrase (1%, enzyme/substrate) or Alcalase (0.15%) was performed at pH 8 (1 N NaOH) and a temperature of 50 °C under continuous stirring (200 rpm). Upon the addition of the enzyme, the pH was maintained at 8 (1 N NaOH) every 15 min, and the volume of the added base was recorded each time for 120 min. The enzymes were inactivated by heating the mixture at 95 °C for 10 min. A combined enzymatic hydrolysis was performed by hydrolysis at half of the time by one enzyme and the addition of the second enzyme after the inactivation of the first enzyme. After cooling, the hydrolysates were centrifuged at 5000*g* for 30 min, and the supernatant was lyophilized and then stored at −80 °C until further use.^22^ The DH was calculated according to the method proposed by Adler-Nissen et al.^23^ as given in eq 1
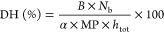
1where *B* is the volume of NaOH consumption in L, *N*_b_ is the normality of the base; MP refers to the mass of protein in kg (nitrogen content × 5.18 for hazelnut^18^), *h*_tot_ is the total number of peptide bonds in the protein substrate (meq/g protein) (the average *h*_tot_ = 8 meq/g for most of the proteins^24^), and α is the average degree of dissociation of the α-amino groups released during hydrolysis expressed as:
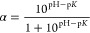
2where p*K* is the average p*K* of the α-NH_2_ groups liberated during hydrolysis, which was assumed to be 8 for hazelnut proteins.^25^ The pH at which the proteolysis was conducted was 8, making α = 0.5 and 1/α = 2.

The following obtained samples were characterized as described in the next sections: PI, hazelnut protein isolate; MFPI, microfluidized PI; NH, Neutrase hydrolysates of PI; MFNH, Neutrase hydrolysates of MFPI; AH, Alcalase hydrolysates of PI, MFAH, Alcalase hydrolysates of MFPI; MFNAH, hydrolysates obtained with sequential hydrolysis, respectively by Neutrase and Alcalase of MFPI; MFANH, hydrolysates obtained with sequential hydrolysis, respectively by Alcalase and Neutrase of MFPI.

### 2.5 SDS-PAGE Analysis

Mini-protean TGX 4-20% precast gels (Bio-Rad Laboratories, USA) were used to perform SDS-PAGE in accordance with Laemmli’s^26^ methodology. The range of the protein molecular standard for this gel was 5–250 kDa. For the range of 5–50 kDa, Mini-protean Tris-Tricine 10-20% precast gels (Bio-Rad Laboratories, USA) were also employed. Briefly, 20 μg of protein was loaded into each well after the loading dye and samples were mixed by 1:1 (v:v). Coomassie Brillant blue G-250 was used to stain protein bands.

### 2.6 Molecular Weight (MW) Distribution

A high-performance size-exclusion chromatography (HP-SEC; Dionex HPLC, Dionex GmbH) outfitted with two serially connected Agilent columns, Agilent Bio SEC-5 (5 μm particle size and 100 Å pore size) and Agilent Bio SEC-5 (5 μm particle size and 300 Å pore size), as well as a UV detector, was used to analyze the hazelnut protein isolates and hydrolysates. A calibration curve of known molecular weights as a function of retention time was made using a commercial protein standard mix with a molecular weight range of 1–670 kDa. Three independent injections (*n* = 3) were used, and the average area of each peak was calculated and displayed in relation to the overall peak area.

### 2.7 Amino Acid Profile

After mixing a known quantity of freeze-dried and powdered samples with 4 mL of 6 N HCl and then flushing with nitrogen gas for 30 s, the process of hydrolysis involved maintaining tubes at 110 °C for 24 h, and the solutions were filtered (using a syringe filter made of PES, 0.2 μm). Samples were diluted before being determined to contain AA using LC/APCI-MS. A 250 × 4.6 mm × 3 μm C18 Phenomenex column connected to an Agilent 6120 quadrupole operating in SIM positive mode was used to inject a 2 μL sample into an LC-MS system (Agilent 1100 HPLC, Waldbron, Germany) for mass spectrometry (Agilent Technologies, Germany). Agilent Mass Hunter, Qualitative software was used to quantify the peak area and compare it to an AA standard mix (ref no. NCI0180. 20,088, Thermo Scientific Pierce, Rockford, IL, USA).

### 2.8 Fourier-Transform Infrared Spectroscopy (FTIR)

An FTIR Tensor II spectrometer was used to determine the secondary structure of the materials (Bruker Optics Inc., Billerica, MA, USA). Lyophilized materials at room temperature were used to determine the spectra as absorbance at wavelengths between 400 and 4000 cm^–1^ in every four scans. After baseline correction, the amide I region (1600–1700 cm^–1^) was smoothed by the Savitzky–-Golay algorithm. The peak was deconvoluted using a Gaussian peak-fit model, and the limit of error (*R*^2^) was converged between 0.99 and 1 by using Origin Pro 2022b software (Origin Lab Corporation, USA). Relative percentages of the peaks corresponding to β-sheet (1610–1645 and 1680–1700 cm^–1^), random coil (1648 ± 2 cm^–1^), α-helix (1652 ± 2 cm^–1^), and β-turn (1660–1680 cm^–1^) were calculated by the area of the deconvoluted peaks.^5^

### 2.9 Scanning Electron Microscopy (SEM)

The surface morphologies of lyophilized protein samples were examined by SEM (ThermoFisher ChemiSEM Axia). A thin layer of platinum was sputter-coated onto the samples before being photographed at a 7.5 kV acceleration voltage and a 100 μm scale with low-vacuum mode (Low Vacuum Detector, LVD).

### 2.10 Dynamic Light Scattering Analysis

The average protein particle size, their polydispersity, and ζ potential were measured with a dynamic light scattering analyzer (Nano-ZS, Malvern Instruments, Worcestershire, UK). Samples were dissolved or diluted to a final concentration of 0.1% in phosphate-buffered saline (PBS) (pH 7.4). The following constants were set: sample refractive index, 1.33; sample absorption: 0.1.^27^

### 2.11 Determination of Emulsifying Properties

The emulsifying properties were determined according to the method by Pearce and Kinsella.^28^ For each sample, 10 mL of olive oil was mixed with 30 mL of protein in PBS solution (3.2 mg/mL). The mixture was homogenized using an Ultra Turrax homogenizer (T18 digital, IKA, Germany) at a speed of 20000 rpm for 1 min. An aliquot of the emulsion (50 μL) was pipetted from the bottom of the container at 0 and 10 min after homogenization and mixed with 5 mL of sodium dodecyl sulfate (SDS, 0.1%) solution. The absorbance of the diluted solution was measured at 500 nm using a UV–vis spectrophotometer (Optima SP-3000 nano spectrophotometer, Tokyo, Japan). The absorbances measured immediately (*A*_0_) and 10 min (*A*_10_) after emulsion formation were used to calculate the emulsifying activity index (EAI) and the emulsion stability index (ESI), as shown in eqs 3 and 4, respectively^29^
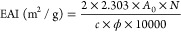
3
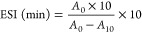
4where *N* refers to the dilution factor, *c* refers to the concentration of the proteins (g/mL), and ϕ refers to the volumetric fraction of the oil (0.25).

### 2.12 Assessment of Free Fatty Acid Release Inhibition

The potential antiobesity activity of hazelnut protein peptides (hydrolysates) was assessed through their ability to inhibit PL, by measuring the amount of free fatty acids (FFA) released from triglycerides. The experimental procedure was performed according to Zhang et al.^30^ with slight modifications. Briefly, a volume of olive oil was homogenized with three volumes of bile salt (1.0 mg/mL) using the Ultra Turrax homogenizer (T18 digital, IKA, Germany). The emulsified substrate (4 mL) was mixed with 1 mL of each peptide solution at different concentrations (4, 8, 16, and 32 mg/mL) or PBS as a control (pH 7.4) and homogenized at 15000 rpm for 2 min. PL (1 mL, 1,6 mg/mL)^31^ previously preincubated at 37 °C for 5 min was added to the mixture to undergo lipolysis for 30 min. The reaction was terminated by the addition of 15 mL of ethanol (95%) and vortexing. The FFA produced was calculated after titration with 0.05 M NaOH to neutralization (pH 7). The inhibition of FFA release (%) was estimated using eqs 5 and 6(32)
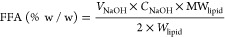
5
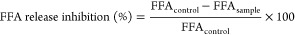
6where FFA (% w/w) corresponds to the amount of free fatty acids released after the digestion process, *V*_NaOH_ is the volume of NaOH (in L) used to neutralize the FFAs released after hydrolysis (assuming that all triacylglycerides are hydrolyzed into two molecules of FFAs and one molecule of monoacylglyceride), *C*_NaOH_ is the molarity of NaOH, MW_lipid_ is the average molecular weight of olive oil (885.4 g/mol) that was deduced from the olive oil composition (99% triglycerides with 80% oleic acid, which refers to glyceryl trioleate),^33^ and *W*_lipid_ is the weight of 1 mL of olive oil (0.917 g), FFA_control_ is the amount of FFA released in the control group, and FFA_sample_ is the amount of FFA released in the sample group.

### 2.13 *In Vitro* Antioxidant Activity

Two different methods assessed the total antioxidant capacity: DPPH and ABTS analyses were performed. The DPPH radical scavenging activity was determined using a modified version of the method described by Wang et al.^34^ Each sample was mixed with 2 mL of 0.1 mM DPPH in a 95% ethanol solution. The mixture was mixed and left for 30 min at room temperature in the dark before its absorbance was measured at 517 nm. The 50% inhibitory concentration values (IC_50_) were calculated using a nonlinear fit to the experimental data and used to evaluate the scavenging activity. The DPPH radical scavenging rate was calculated using eq 7.
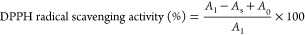
7where *A*_0_ represents the absorbance of ethanol and the sample solution at 517 nm, *A*_1_ represents the absorbance of ethanol and DPPH at 517 nm, and *A*_s_ represents the absorbance of the sample solution and DPPH at 517 nm.

The ABTS radical scavenging activity was determined following the method of Liu et al.^35^ with slight modifications. First, ABTS and potassium persulfate were combined after being dissolved in distilled water to final concentrations of 7 and 2.6 mmol/L, respectively. The mixture was allowed to remain dark at room temperature for 12 h before use. It was then diluted by combining 1 mL of ABTS solution with 60 mL of PBS to get an absorbance of around 1.00 at 734 nm using a spectrophotometer. PBS was used to dissolve all analyzed samples. A 5 mL portion of fresh ABTS solution was mixed with 500 L of analyzed materials for 2 h in the dark. A spectrophotometer was then used to measure the absorbance at 734 nm. PBS was used as a blank control.

The ABTS radical scavenging activity was calculated according to the eq 8
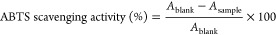
8

### 2.14 Statistical Analysis

All analyses were performed in triplicate, and the data were presented as mean ± standard deviation (SD), one-way analysis of variance (ANOVA) followed by Duncan’s post hoc multiple comparison test using GraphPad Prism 8.0 (San Diego, California, USA) and the SPSS statistics program (Version 28.0) for IOS (SPSS Inc. Chicago, IL).

## 3 Results and Discussion

### 3.1 Protein Hydrolysis Efficiency

#### 3.1.1 Degree of Protein Hydrolysis and SDS-PAGE Patterns

**Figure 1:**
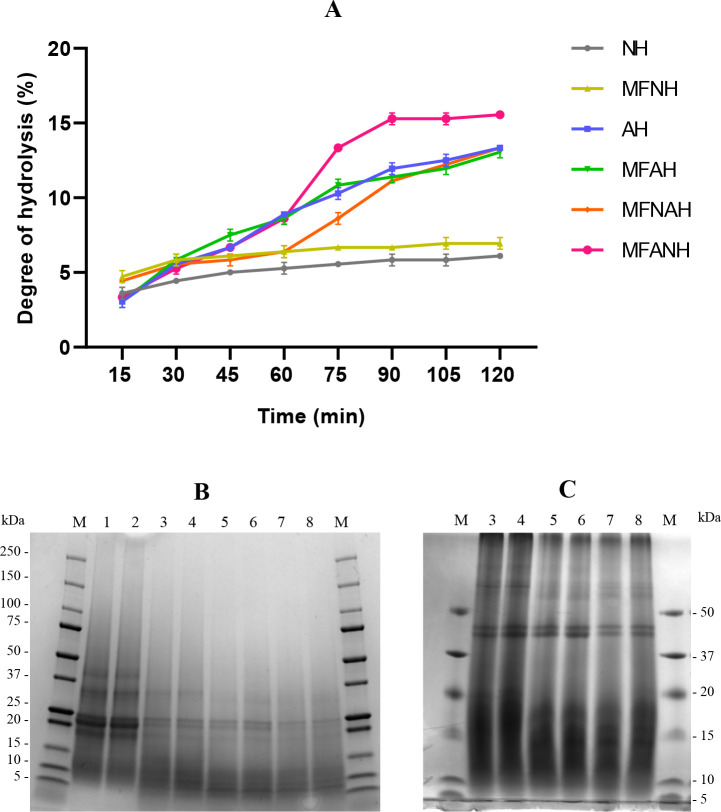
Degree of hydrolysis (A) and changes in SDS-PAGE profiles of microfluidized and nonmicrofluidized hazelnut protein isolates and their hydrolysates, prepared with Alcalase, Neutrase, or the combination of both (B, C): M, marker; 1, PI; 2, MFPI; 3, NH; 4, MFNH; 5, AH; 6, MFAH; 7, MFNAH; 8, MFANH.

The combined enzymatic hydrolysis showed sigmoidal curves, while individual enzymes showed the usual asymptotic curves (Figure 1A). The highest DH was achieved by an Alcalase–Neutrase combined enzymatic treatment (MFANH) with a maximum of 15.57 ± 0.0%. When the order of the enzyme treatment was inverted (MFNAH), the DH dropped to 13.34 ± 0.0%, which was not significantly different from that of an Alcalase single enzymatic treatment (AH: ,13.34 ± 0.0%; MFAH, 13.06 ± 0.39%). Neutrase showed the lowest DH with values of 6.12 ± 0.0% (NH) and 6.95 ± 0.39% (MFNH). Differences between microfluidized and nonmicrofluidized samples were not significant.

The SDS-PAGE profiles confirm the different hydrolysis rates achieved by each sample (Figures 1B,C). Protein isolates (PI and MFPI) exhibited five distinct protein patterns: 1, 37–50 kDa; 2, 25–37 kDa; 3, 25 kDa; 4, 20–25 kDa (the most intense protein); 5, 15–20 kDa (Figure 1B). The intensity of these protein bands was significantly reduced upon enzymatic hydrolysis, with the most reduction shown by each combination of Alcalase and Neutrase (MFNAH and MFANH).

It has been reported that microfluidization pretreatment might enhance the hydrolysis of specific proteins by different enzymes: e.g., soy protein by pancreatin^21^ and papain^36^ and oyster protein by trypsin.^37^ The insignificant effect of microfluidization on the DH of hazelnut protein in our study could be due to an insignificant change in the total available cleavage sites for Alcalase and Neutrase, despite the observed morphological and functional changes described in the following sections. The literature lacks sufficient information on the effect of microfluidization on the hydrolysis of proteins by these two enzymes. Zhang et al.^38^ studied the effect of microfluidization on the functional properties of rice dreg protein before hydrolysis by Alcalase and Neutrase, but the authors did not provide details on the changes in the DH. A combined enzyme hydrolysis of proteins may result in a synergistic effect on both the DH and the biological activities of the hydrolysates.^39−42^ Nevertheless, research on this promising strategy is still very limited and needs to be further explored. Alcalase and Neutrase are bacterial endoproteases produced by *Bacillus licheniformis* and *Bacillus amyloliquefaciens*, respectively. The first is a relatively aggressive but stereoselective serine protease, while the latter is a mild metalloprotease (Novozymes catalogs). Wang et al.^43^ found that the sequential hydrolysis of whey protein with Alcalase and Neutrase was more efficient and effective in inhibiting ACE activity than a single-enzyme hydrolysis or an Alcalase–trypsin combination.

### 3.2 Molecular Weight (MW) Distribution of Protein Isolates and Hydrolysates

**Figure 2:**
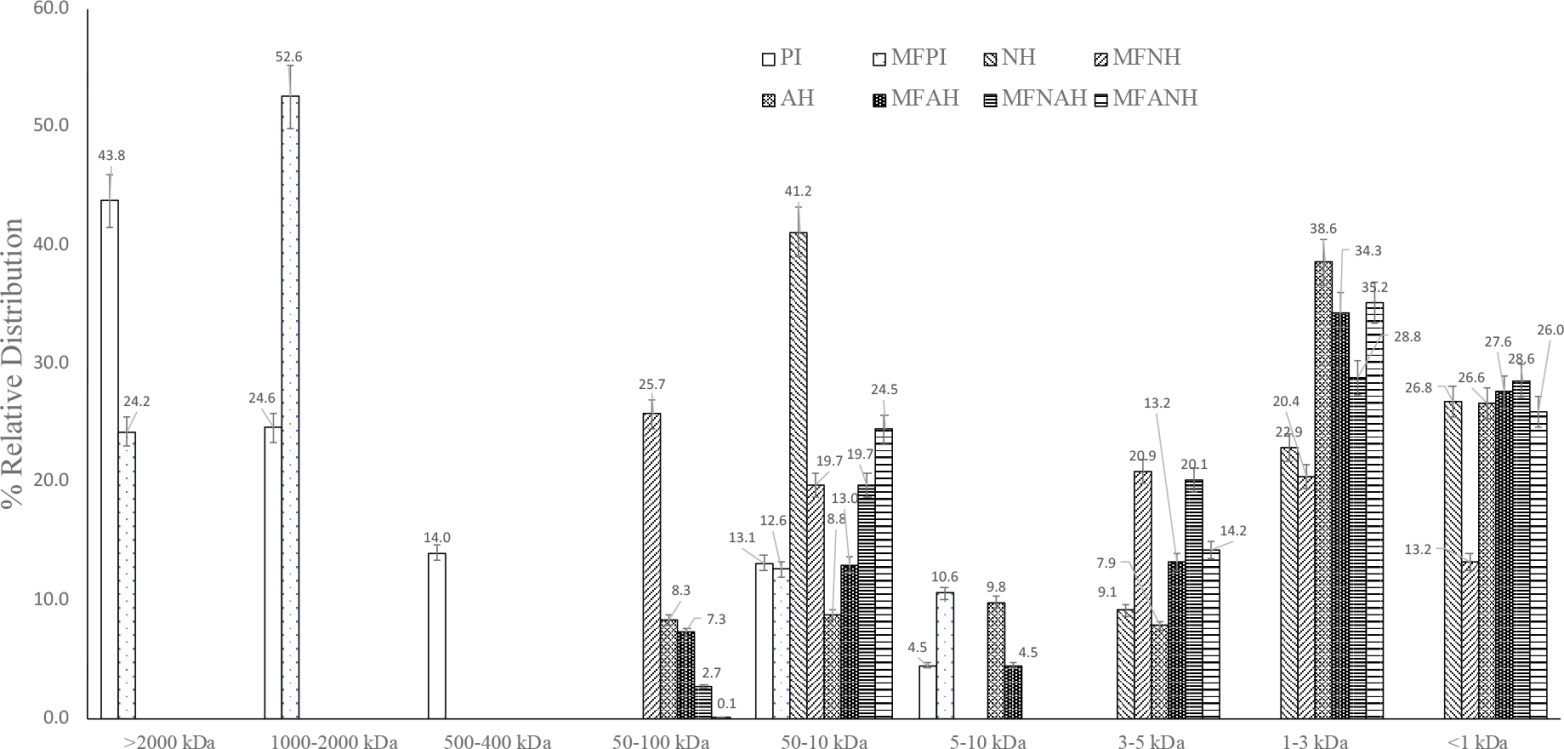
Molecular weight distribution of hazelnut protein isolates and their hydrolysates, prepared with Alcalase, Neutrase, or a combination of both.

The molecular weight profiles of microfluidized and nonmicrofluidized hazelnut protein isolates and their hydrolysates, prepared with Alcalase, Neutrase, or a combination of both, are displayed in Figure 2.

The pattern of the MW distribution of PI was >2000 kDa (43.8%), 1000–2000 kDa (24.6%), 400–500 kDa (14.0%), 10–50 kDa (13.1%), and 5–10 kDa (4.5%). The MW distribution increased 52.6% at 1000–2000 kDa, while more (10.6%) peptides were obtained at 5–10 kDa with the microfluidization pretreatment. In contrast, a microfluidization pretreatment negatively affected Neutrase hydrolysates. Peptide fractions obtained by Neutrase hydrolysis were distributed at <50 kDa; however, larger-size peptide fractions (50–100 kDa) were obtained by Neutrase hydrolysis with microfluidization pretreatment. There was no significant effect of microfluidization on Alcalase hydrolysates. On the other hand, peptide fractions were extensively obtained at <3 kDa with the combined enzyme treatments (MFNAH and MFANH).

The molecular weight is a crucial factor that reflects protein hydrolysis and corresponds with the bioactivity of protein hydrolysates.^44^ AH and MFAH had high percentages of a <3 kDa fraction, while lower percentages of this fraction were found in NH and MFNH. This revealed that this fraction was a significant substrate for Alcalase.

### 3.3 Amino Acid Profile of Hazelnut Protein Isolates and Hydrolysates

The amino acid composition of hazelnut meal protein isolate was analyzed in order to assess its potential as a functional food component. The results are shown in Table 1.

**Table 1:** Amino Acid Profile of Hazelnut Meal Protein Isolates (mg/g Protein)^a^

Amino acid	Content	Amino acid	Content
LYS	11.04 ± 0.7	MET	6.44 ± 0.7
ARG	83.22 ± 3.5	TYR	21.4 ± 0
HIS	17.12 ± 1.4	ILE	51.18 ± 1.7
GLY	28.81 ± 0.7	LEU	51.02 ± 3.4
SER	33.92 ± 0.3	PHE	34.03 ± 1.8
ALA	32.24 ± 0.9		
THR	23.42 ± 0.9		
GLU	80.34 ± 0.3	∑ΕΑΑs	216.81 ± 11.7
ASP	151.75 ± 0.7	∑ΒCΑΑs	131.21 ± 6.8
PRO	25.92 ± 0.5	∑ΝΕΑΑs	298.26 ± 2.3
VAL	29 ± 1.7		

a The data are presented as the mean ± standard deviation of three replicates. Abbreviations: LYS, lysine; ARG, arginine; HIS, histidine; GLY, glycine; SER, serine; ALA, alanine; THR, threonine; GLU, glutamic acid; ASP, aspartic acid; PRO, proline; VAL, valine; MET, methionine; TYR, tyrosine; ILE, isoleucine; LEU, leucine; PHE, phenylalanine, EAAs, essential amino acids; BCAA, branched-chain amino acids; NEAA, nonessential amino acids.

Aspartic acid (151.75 ± 0.7 mg/g), arginine (83.22 ± 3.5 mg/g), and glutamic acid (80.34 ± 0.3 mg/g) were the major amino acids found in PI. The essential amino acid (EAA) contents of PI that are used as functional food ingredients are crucial, especially in terms of sports nutrition,^45^ since these amino acids act as substrates in muscle protein synthesis.^46,47^ The EAA content of PI was determined to be 32.79% of total amino acids, and leucine and isoleucine were the major EAAs. This result was similar to the result of Sen and Kahveci^47^ and was substantially higher than those for many different plant-based proteins such as lupin (21%), oat (21%), wheat (22%), hemp (23%), soy (27%), and brown rice (28%).^46^ On the other hand, branched-chain amino acids (BCAAs) have also been reported to be potent stimulators of skeletal and liver protein synthesis in addition to serving as substrates.^45^ Thus, the BCAA content (sum of leucine, isoleucine ,and valine) of PI was also determined and found to be 19.27% of total protein in PI, which was higher than the results of Sen and Kahveci^47^ and for other plant-based proteins mentioned above. Moreover, the nonessential amino acid (NEAA) content was found to be 67.21% of the total protein in PI.

### 3.4 Fourier-Transform Infrared (FTIR) Spectroscopy

FTIR is a method that provides information about the structures of proteins and measures the wavelength and strength of IR radiation’s absorption.^48^ FTIR measures the vibrational transitions of functional groups, generally between the ground state and the first excited state (4000–500 cm^–1^). Covalent bonds in atoms exhibit distinctive vibrations known as stretching (changes in bond lengths) and bending (changes in bond angles).^49^ To make assumptions about the presence of a specific functional group of the molecule, the FTIR spectrum is divided into four distinct regions according to their binding type: single bond (O–H, C–H, N–H: 2500–4000 cm^–1^), triple bond (C≡C, C≡N, X=C=Y: 2000–2500 cm^–1^), double bond (C=O, C=N, C=C: 1500–2000 cm^–1^), and fingerprint region (500–1500 cm^–1^).^50^

**Figure 3:**
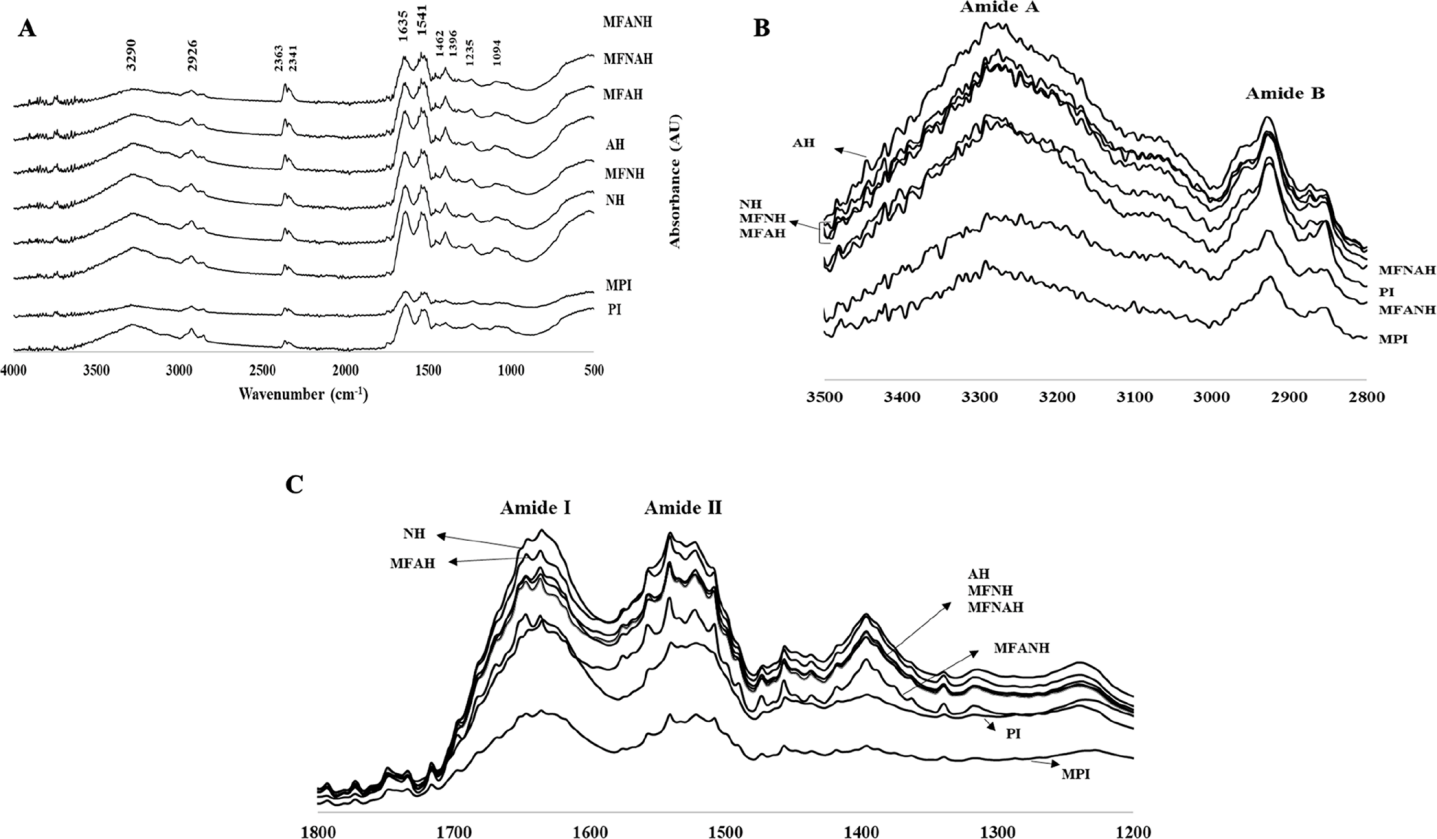
FTIR spectra of hazelnut protein isolates and their hydrolysates, prepared with Alcalase, Neutrase, or the combination of both in the ranges of 500–4000 cm^–1^ (A), 2800–3500 cm^–1^ (B), and 1200–1800 cm^–1^ (C).

FTIR spectra of all protein samples are given in Figure 3. Nine distinct IR peaks of protein concentrate were observed by the various functional groups from lipids, proteins, and other substances. These are amide A, amide B, amide I, amide II, and fingerprint region bands (Amides III–VII). The maximum intensity points of PI for these bands are indicated in Figure 3. Microfluidization and enzyme treatment change the conformational properties of proteins. Although there was a change in the intensity of the peaks after different treatments, no new peak was observed in any sample. The most prominent peaks were observed between 1200 and 1800 cm^–1^ and betweeen 2800 and 3500 cm^–1^.

Two peaks determined in the fingerprint region of the native protein PI originated from bending vibrations of aliphatic CH_2_ at 1462 cm^–1^ (asymmetrical) and at 1396 cm^–1^ (symmetrical).^51^ The bands at 1233 and 1094 cm^–1^ are antisymmetrical and symmetrical double-bond stretching vibrations of the phosphate moiety (PO_2_^–^), respectively.^52^ All of the bands in the fingerprint region are specific to the molecule and are often used to describe the molecules.

Moreover, Kavipriya and Ravitchandirane^53^ stated that the peaks at 2363 and 2341 cm^–1^ in the triple-bond region were related to CO_2_ symmetrical axial deformation, and these peaks in this region were ignored in this study.

The peaks between 1200 and 1800 cm^–1^ and between 2800 and 3500 cm^–1^ were the most significant ones. The spectrum of PI had a characteristic amide A band at 3290 cm^–1^ (tensile vibration of intermolecular hydrogen bonding between O–H and N–H stretching occurring in the hydrogen bonds and intermolecular H bonding), amide B bands at 2926 cm^–1^ (asymmetric stretching of aliphatic CH_2_) and 2854 cm^–1^ (symmetrical stretch of aliphatic CH_2_), amide I band at 1635 cm^–1^ (C=O stretching vibrations of peptide linkages), and amide II at 1541 cm^–1^ (C–N stretching, 40%, and N–H bending, 60% of amino acids).^51^ The changes in the spectra in the range of 2800–3500 cm^–1^ are indicated in Figure 3B. An increase in the intensity of the amide A band indicates the formation of hydrogen bonds and a decrease in the hydrogen bonds indicates that the hydrogen bonds are partially broken down.^54^ MFANH and MFPI had lower peak intensities while the other protein samples had higher intensities. However, the amide B bands (2800–3000 cm^–1^) of MFANH and MFPI were lower than those of PI and were blue-shifted to 2924 and 2852 cm^–1^, which means that the hydrophobic regions of the protein (aliphatic groups) were destroyed.^54^ An increase in the intensity of the peaks in this region is related to the fragmentation of the proteins into smaller pieces as a result of the enzyme application and the exposure of their hydrophobic regions.

The two major peaks (amide I and II) are clearly related to the structure of the peptide bond (OC–NH). Since the peak intensities in the protein backbone region (800–1800 cm^–1^) are related to the vibrations of the peptides in the sample, they vary linearly with the decomposition products of the protein. Therefore, the area under these bands was calculated in Table 2 to see the difference in treatments in contrast to the control (PI).^55^ The secondary structure of the proteins was calculated by the deconvoluted amide I band in Table 2.

**Table 2:** Secondary Structure Content (%) of Protein Samples from FTIR Data^a^

	PI	MPI	NH	MFNH	AH	MFAH	MFNAH	MFANH
Integrated Areas								
amide I(1712–1579 cm^–1^)	5.28 ± 0.5	2.82 ± 0.9	7.37 ± 0.6	6.32 ± 0.1	5.95 ± 0.6	6.49 ± 0.9	5.70 ± 0.7	4.80 ± 0.4
amide II(1579–1477 cm^–1^)	2.67 ± 0.3	1.56 ± 0.08	4.33 ± 0.2	3.69 ± 0.3	3.52 ± 0.08	4.03 ± 0.4	3.60 ± 0.7	3.10 ± 0.03
amide A(3700–3000 cm^–1^)	16.69 ± 0.2	7.87 ± 0.6	20.31 ± 0.06	18.91 ± 0.5	22.72 ± 0.2	17.98 ± 0.7	15.43 ± 0.2	9.29 ± 0.1
amide B(3000–2815 cm^–1^)	2.26 ± 0.1	1.30 ± 0.5	1.84 ± 0.1	2.09 ± 0.2	2.18 ± 0.3	1.97 ± 0.7	1.83 ± 0.1	1.27 ± 0.3
Secondary Structure (%)								
α-helix	0.86	0.00	26.21	57.51	39.13	65.56	83.08	50.99
β-sheet	55.98	47.46	34.80	22.98	25.22	17.45	14.31	34.53
β-turn	20.35	19.76	19.29	15.81	27.20	15.63	0.15	0.22
random coil	22.81	32.78	19.71	3.69	8.45	1.36	2.46	14.26
α-helix +β-sheet	56.83	47.46	61.01	80.49	64.35	83.01	97.39	85.52

a The data are presented as the mean ± standard deviation of three replicates.

After the microfluidization process (MFPI), a significant decrease in the intensity of the amide A, I, and II bands and even the disappearance of the peaks were observed. It has been stated that, after the microfluidization process, the amide I band density of rice dreg protein^6^ and peanut protein^5^ decreased and unfolded in the structures of the proteins, especially due to the increase in the random coil structure. Microfluidization is an efficient pretreatment technique that aids in enzymatic hydrolysis. Pressure changes the protein structure and affects the enzyme’s cleavage sites, changing the rate of hydrolysis and the properties of the protein.^6^

Since the sum of α-helix and β-sheet gives the total intermolecular hydrogen bonds (degree of compactness),^54^ they are also indicated in Table 2. The β-sheet structure in all protein samples compared to PI was decreased and the α-helix structure was increased especially after enzyme treatments, similar to the Alcalase hydrolysis of potato protein isolate reported by Akbari et al.^56^ β-Sheet structures, which preserve hydrophobic amino acids, are in the interior regions of folded proteins, while α-helix structures typically reside on the exterior of protein molecules. As a result of the protein unfolding during hydrolysis, (1) the β-sheet structure disassembled and decreased by the revealing of hidden hydrophobic residues and (2) an improvement in the α-helix structure of the protein was observed.^56^

A significant increase was observed in the amide A band (hydrogen bonding was intense) and amide B (aliphatic groups were exposed) in the enzyme application alone (AH and NH), while a decrease in the amide A and B bands was observed with the microfluidization application beforehand (MFAH and MFNH).

For MFNAH and MFANH, there was less intensity in the amide A, B, and I and II bands compared to the enzyme treatments alone (MFNH and MFAH). Since Neutrase is more selective than Alcalase, the first use of Alcalase may have increased the number of free amino acids transferred to the water phase and increased the availability of amino acids for the more selective Neutrase (MFANH). Consequently, applying microfluidization before enzyme application or using first Alcalase and then Neutrase enzyme caused a decrease in these amide bands.

### 3.5 Scanning Electron Microscopy (SEM)

**Figure 4:**
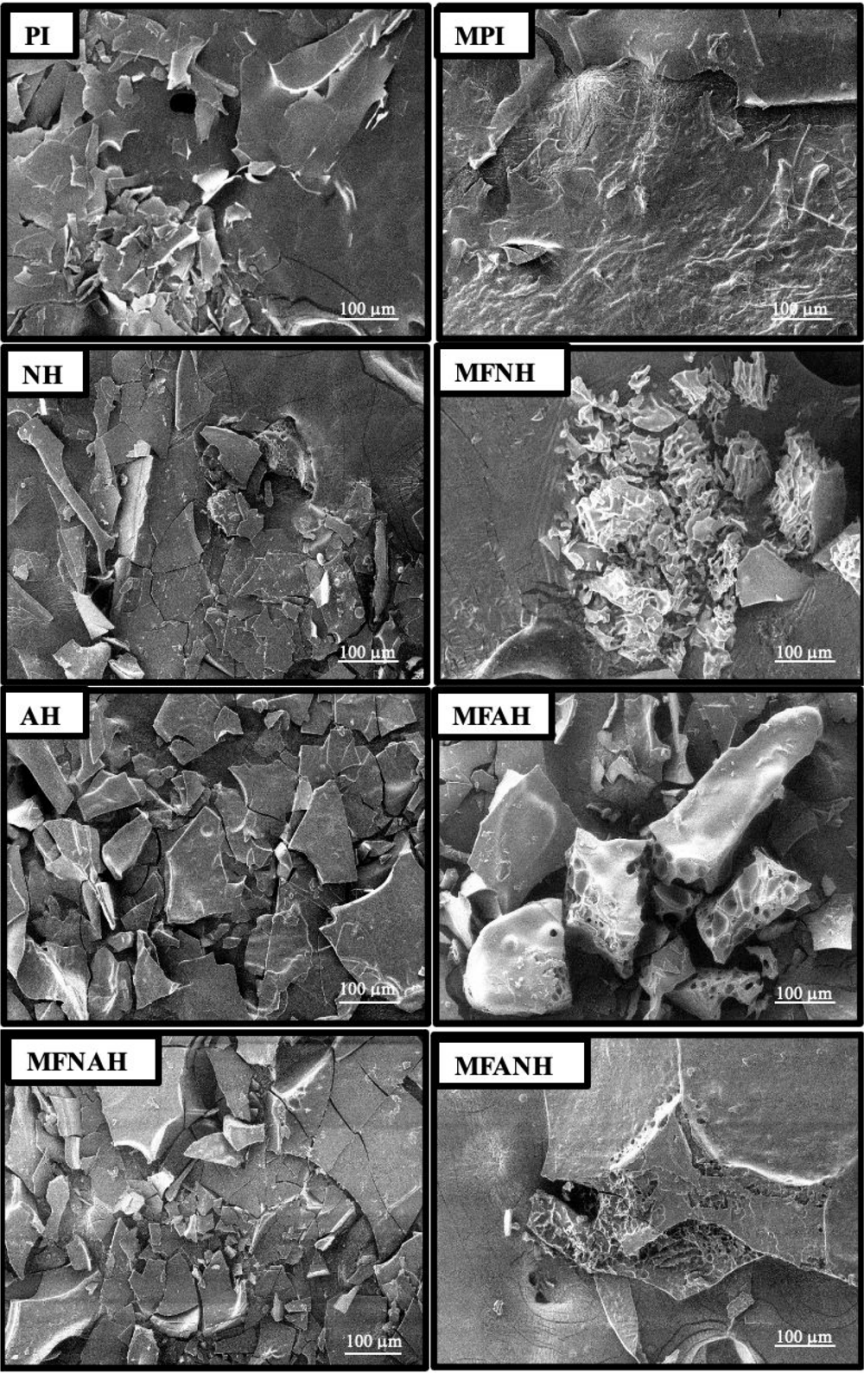
Morphologies of microfluidized and nonmicrofluidized hazelnut protein isolates and their hydrolysates, prepared with Alcalase, Neutrase, or the combination of both.

A microfluidization or enzyme treatment caused a reduction in the particle size (Figure 4) of the proteins. The microstructure of native protein (PI) is in the form of heterogeneous large clumps with a flakelike and smooth structure. This was probably due to the removal of water during the lyophilization process.^57^ The absence of forces necessary to break up the frozen liquid into droplets or significantly modify their surface topology during the freezing evaporation process may be the cause of the bigger particles and flakelike shape.^58^ A significant change in the surface structure of the protein was observed with the deformation of the proteins by the microfluidization treatment. Massive, irregular protein clumps were broken up into smaller sizes, in line with the results obtained by particle size analysis. Similarly, large clumps of rice dreg protein,^6^ fenugreek,^57^ and peanut protein^5^ were reported to be fragmented into smaller clumps after the microfluidization, together with a decrease in particle size. Enzyme treatment alone (Alcalase or Neutrase) supported the formation of low-sized clumps on the one hand, and large clumps were formed on the other. More platy, fragile, and angular structures were observed in AH than in NH. The enzyme treatment together with microfluidization supported the formation of small clumps in the samples. A similar result was obtained by Hu et al.^5^ with the application of microfluidization and *trans*-glutaminase enzyme of peanut proteins. Proteins with a lower molecular structure may come into contact with more water, causing the structure to become more porous when the water sublimes. This is evident in MFANH, which has a significantly smaller pore structure.

### 3.6 Particle Size, Size Distribution, and ζ Potential

**Figure 5:**
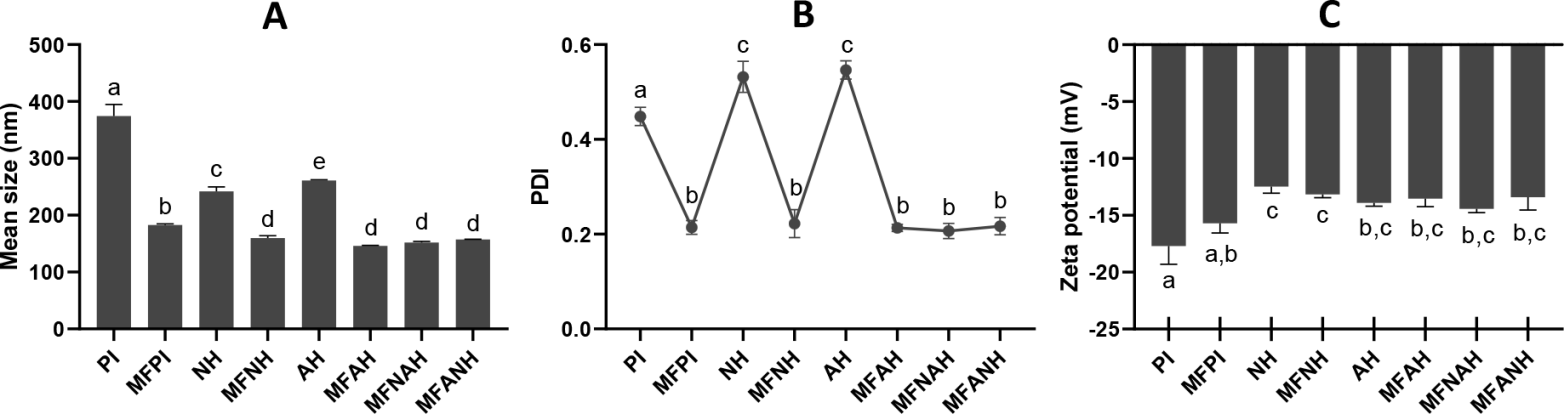
Average particle size (A), size distribution (B), and surface charge (C) of microfluidized and nonmicrofluidized hazelnut protein isolates and their hydrolysates, prepared with Alcalase, Neutrase, or the combination of both. Lower-case letters (a–d) indicate statistically significant differences (*P* < 0.05).

Both microfluidization and enzymatic hydrolysis treatments significantly reduced the average particle size of hazelnut protein isolate, from 374.3 ± 20.65 nm (PI) to 182.6 ± 2.26 (MFPI), 241.97 ± 7.75 (NH), and 261.13 ± 0.96 nm (AH). The smallest size was observed in proteins that have undergone both microfluidization and proteolysis, with no significant differences among these samples: 159.9 ± 4.23 (MFNH), 145.90 ± 0.85 (MFAH), 151.97 ± 2.07 (MFNAH), and 157.1 ± 0.44 nm (MFANH) (Figure 5A).

Likewise, microfluidization reduced the size dispersity of the protein particles, from a PDI of 0.45 ± 0.02 (PI) to ≅0.21 for all microfluidized samples, regardless of the enzymatic hydrolysis (Figure 5B). However, nonmicrofluidized hydrolysates showed a slight increase in their PDI: 0.53 ± 0.03 (NH) and 0.55 ± 0.02 (AH). Hazelnut protein isolates and hydrolysates were negatively charged with ζ potentials between −12.47 ± 0.59 and −17.7 ± 1.61 mV (Figure 5C). Particle size and size polydispersity reduction is a common consequence of protein microfluidization.^59,60^ Generally, enzymatic hydrolysis also reduces the size of protein particles.^61,62^

### 3.7 Emulsifying Properties

**Figure 6:**
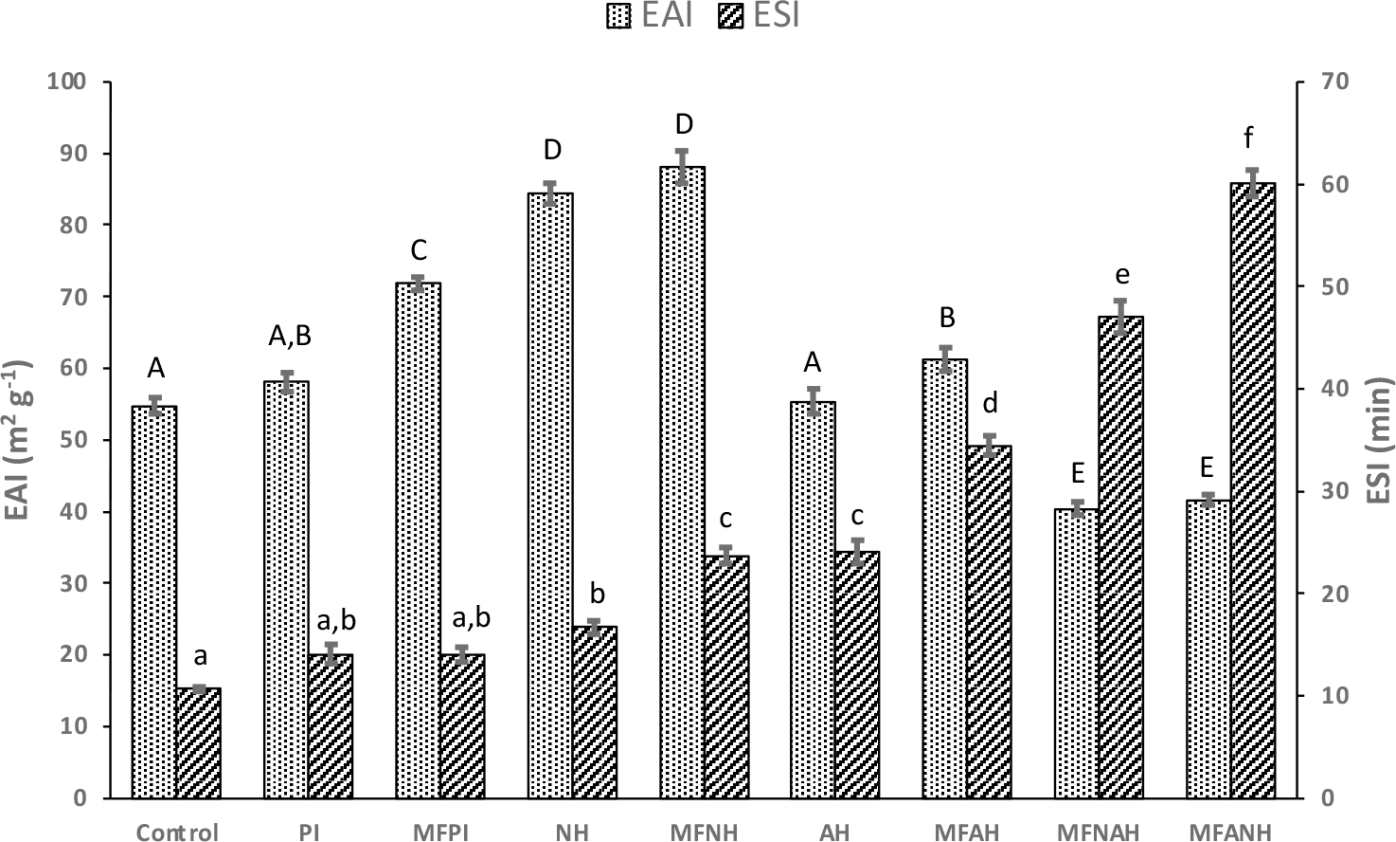
Emulsifying properties of microfluidized and nonmicrofluidized hazelnut protein isolates and their hydrolysates, prepared with Alcalase, Neutrase, or the combination of both. Results are expressed as EAI and ESI. Different lower-case letters (a–f) indicate significant differences between ESI groups (*p* < 0.05). Different capital letters (A–D) indicate significant differences between EAI groups (*p* < 0.05).

Emulsifying properties of microfluidized and nonmicrofluidized hazelnut protein isolates and their hydrolysates, prepared with Alcalase, Neutrase, or the combination of both, are expressed as the EAI and ESI (Figure 6). The emulsifying activity index is expressed as the area of oil/water interface stabilized per unit weight of protein. The emulsion stability index is expressed as the time needed to achieve a turbidity of the emulsion that is half of its original value. Microfluidization treatment allowed a significant increase in terms of EAI, from 58.76 ± 1.34 (PI) to 71.80 ± 0.9 m^2^/g (MFPI). Hydrolysates obtained by Neutrase showed the best emulsifying activity with EAI of 84.32 ± 1.43 (NH) and 88.04 ± 2.22 m^2^/g (MFNH). In contrast, Alcalase hydrolysis did not significantly improve the EAI of the protein isolates, 55.36 ± 1.77 (AH) and 61.24 ± 1.63 m^2^/g (MFAH), even though the EAI in MFAH was significantly higher than that in AH. Interestingly, the combined enzymatic treatment reduced the EAI of the proteins to 40.35 ± 0.94 (MFNAH) and 41.65 ± 0.65 m^2^/g (MFANH), which again correlates with the highest degree of hydrolysis achieved by these samples. Unlike EAI, the best ESI was achieved by the most hydrolyzed proteins, 46.96 ± 1.58 (MFNAH) and 60.03 ± 1.28 min (MFANH).

Alcalase hydrolysates showed better ESI values than Neutrase, which also positively correlates with their respective degrees of hydrolysis. Microfluidization had an additional positive impact on the stability of emulsions in hydrolyzed proteins, 23.70 ± 0.81 (MFNH) vs 16.71 ± 0.64 min (NH) and 34.42 ± 0.91 (MFAH) vs 24.02 ± 1.12 min (AH). Protein isolates showed the lowest ESI values at 14.04 ± 0.94 for PI and 14.00 ± 0.75 min for MFPI, with no significant differences from the control, 10.69 ± 0.21 min.

Similar to our results, Imura et al.^63^ obtained the best emulsifying properties with soybean hydrolysates with the lowest DH and the worst properties with the most hydrolyzed proteins, even lower than those of protein isolates. An increased DH of proteins has been associated with low emulsifying capacity by many other researchers.^64−68^ Inversely, the stability index of emulsions was reported to be higher in hydrolysates with the greatest DH by Baharuddin et al.,^69^ which is in agreement with our present data. However, protein hydrolyzation can also independently increase EAI, decrease ESI, or have no significant effect on the emulsifying properties of proteins.^70−74^ This was reported to be dependent on other factors, including the pH, protein source, processing steps, and the enzyme type being used for the hydrolysis process.^75^ For instance, Tamm et al.^76^ found that a higher DH by Alcalase on pea protein had a negative effect on their emulsifying properties while trypsin improved emulsions with an increase in the DH of the same protein source. Thus, further studies directly determining the effects of structural and surface properties of protein hydrolysates on their functional properties would be very useful.^75^ Recently,^38^ Zhang et al. reported a negative effect of microfluidization (120 MPa) on rice dreg protein isolate on EAI and no effect on ESI, which differs from our findings for hazelnut isolates. This once again shows that the impact of a single parameter can drastically differ when the other parameters are different. Nevertheless, in this study the emulsification conditions were identical among all samples; thus, the differences among samples may only be ascribed to their respective functional properties.

### 3.8 Inhibition of FFA Release

**Figure 7:**
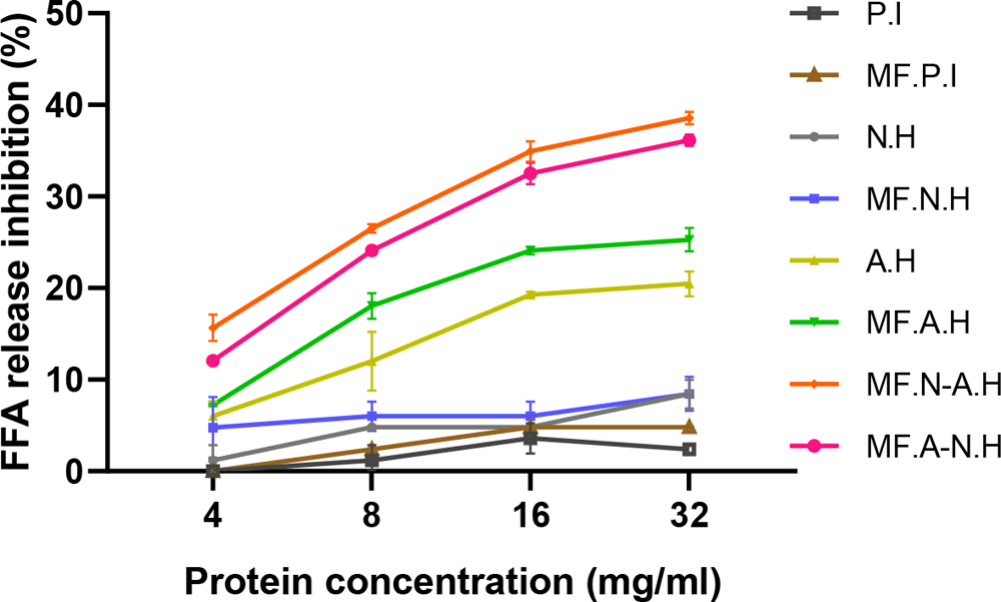
Inhibition of FFA (%) of microfluidized and nonmicrofluidized hazelnut protein isolates and their hydrolysates, prepared with Alcalase, Neutrase, or the combination of both.

Hazelnut protein isolates and hydrolysates showed a dose-dependent inhibition of FFA release by the action of pancreatic lipase on olive oil triglycerides (Figure 7). The best FFA inhibition was achieved by the most hydrolyzed proteins which were obtained by combining Alcalase and Neutrase, i.e., MFNAH and MFANH, with respectively maximum inhibitions of 38.56 ± 0.66% and 36.15 ± 0.62% at a concentration of 32 mg/mL and minimum inhibitions of 15.65 ± 1.44% and 12.05 ± 0.21% at a concentration of 4 mg/mL. There were no significant differences between these two samples. Among Alcalase hydrolysates, microfluidization had a significant positive effect on the inhibition of FFA release in comparison with nonmicrofluidized samples at all test concentrations (at 32 mg/mL: 25.29 ± 1.27% (MFAH) vs 20.47 ± 1.36% (AH)), except for the lowest concentration, which had no significant difference between these two samples (7.23 ± 0.12% and 6.01 ± 1.6%, respectively). Neutrase hydrolysates had lower FFA release inhibition with maximums of 8.45 ± 1.85% (MFNH) and 8.42 ± 1.56% (MH), which correspond to the lowest degrees of hydrolysis. Protein isolates had the lowest inhibition with 4.82 ± 0.08% (MFPI) and 2.41 ± 0.04% at their highest concentrations.

Plant-based bioactive peptides may inhibit PL through different mechanisms. They may bind directly to the active site of the enzyme, alter its conformation, or destroy bile salt/oil emulsions, on which lipase relies for effective hydrolysis, by altering the lipophilic/hydrophilic balance of bile salts.^30^ This last mechanism is in accordance with the low emulsifying activity shown by MFNAH and MFANH in the present study. Wang et al.^77^ identified a bioactive peptide (RLLPH) isolated from *Corylus heterophylla* Fisch. which, in addition to the inhibition of PL, had an adipogenesis inhibition in 3T3-L1 adipocytes by the regulation of adipogenic transcription factors and adenosine monophosphate activated protein kinase (AMPK) activation. This makes hazelnut protein hydrolysates good candidates as functional food ingredients with antiobesity potential.

### 3.9 *In Vitro* Antioxidant Activity of Protein Isolates and Hydrolysates

#### 3.9.1 DPPH Radical Scavenging Activity

**Figure 8:**
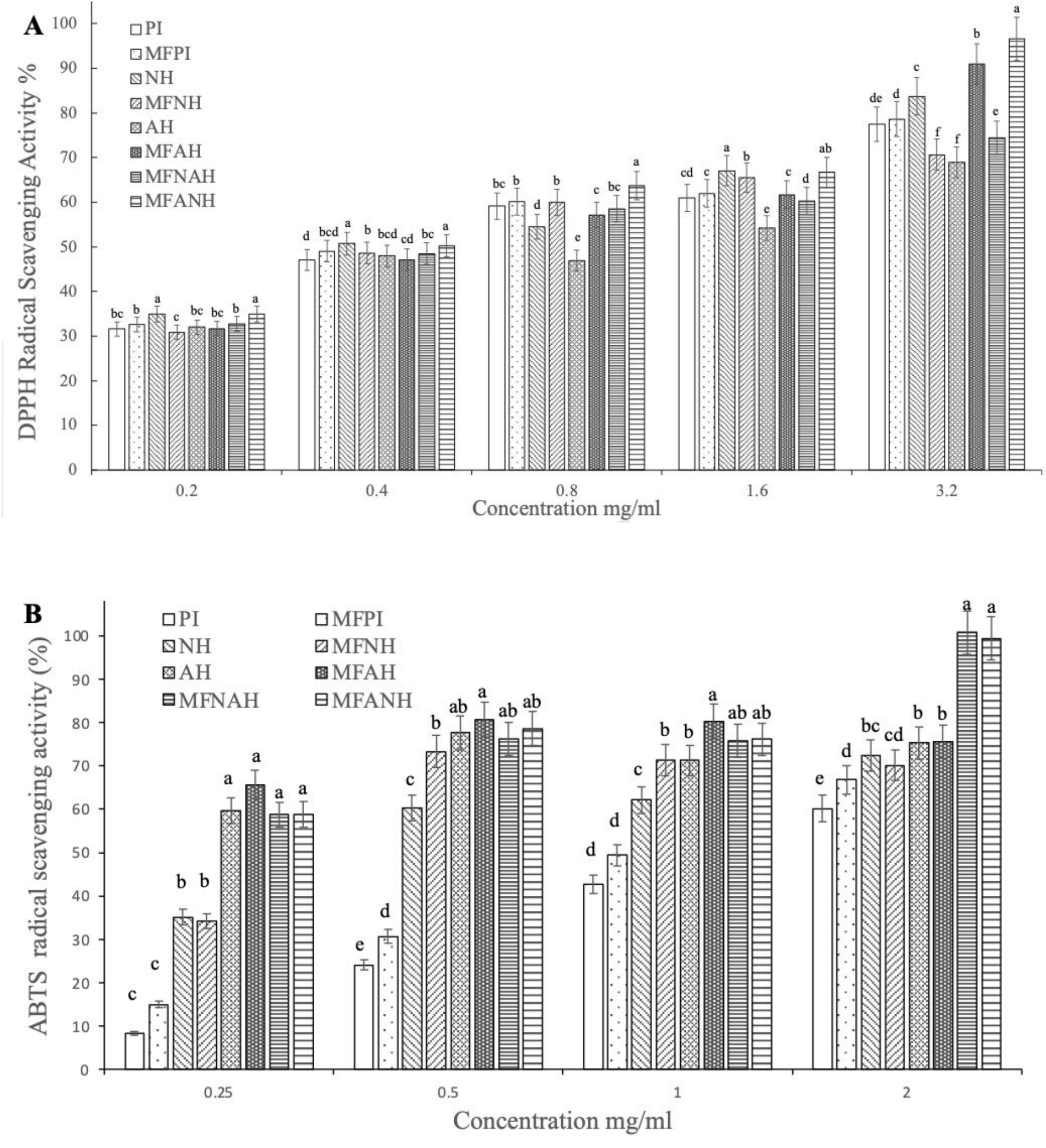
DPPH (A) and ABTS (B) radical scavenging activities of microfluidized and nonmicrofluidized hazelnut protein isolates and their hydrolysates, prepared with Alcalase, Neutrase, or the combination of both. Different lower case letters (a–d) indicate statistically significant differences (*P* < 0.05).

The DPPH radical is frequently used to measure antioxidant activity of numerous natural bioactive compounds due to its stability and potential to behave as a free radical scavenger.^78^ A concentration-dependent analysis was performed on hazelnut PI and hydrolysates prepared with Neutrase and Alcalase with or without microfluidization pretreatment and their combinations. Figure 8A shows the DPPH radical scavenging activity of hazelnut PI and its hydrolysates at various concentrations (0.2–3.2 mg/mL). As shown in Figure 8, the effect of combined enzyme application and microfluidization pretreatment on the DPPH radical scavenging activity becomes increasingly significant as the concentration increases (*p* < 0.05). The DPPH radical scavenging activities of MFNAH and MFANH at the highest concentration (3.2 mg/mL) were 74.45 ± 1.94% and 96.63 ± 1.06%, respectively. Microfluidization pretreatment made no statistically significant difference in the DPPH radical scavenging activity of PI as the concentration increased. In contrast, it negatively affected hydrolysates prepared using Neutrase and positively affected hydrolysates prepared using Alcalase. (*p* < 0.05). The DPPH radical scavenging activities of combined Alcalase–Neutrase hydrolysates with microfluidization pretreatment were higher than those of combined Neutrase–Alcalase hydrolysates with microfludization pretreatment at all concentrations (*p* < 0.05), which indicates the order of the enzyme is critical in combined enzyme application. These results are in accordance with the findings of Zhang et al.^79^ on rice dreg protein isolates. For the hazelnut PI and hydrolysates, the lowest IC_50_ value (the highest radical scavenging activity) of MFANH was 0.86 mg/mL, followed by 0.91, 0.97, 0.98, 1.18, 1.24, 1.25, and 1.53 mg/mL for MFAH, MFNH, NH, MFPI, MFNAH, PI, and AH respectively. The differences in DPPH radical scavenging activities across hydrolysates may be linked to free amino acid compositions, molecular distribution, peptide structures, and sequencing.^80,81^

#### 3.9.2 ABTS Radical Scavenging Activity

The peroxidase substrate ABTS has become a useful substrate for assessing total antioxidant capacity, since it forms a relatively stable radical (ABTS) on one-electron oxidation.^35,82^ The ABTS radical scavenging activity of the hazelnut PI and hydrolysates were measured at 0.25–2 mg/mL to further confirm the antioxidant activities, and the results are shown in Figure 8B. Increasing trends were monitored for the ABTS radical scavenging activities of PI and MFPI in a concentration-dependent manner. No significant difference was observed in the radical scavenging activities of individual enzyme hydrolysates with or without microfluidization pretreatment (NH, MFNH, AH, MFAH) after 0.5 mg/mL concentration. Unlike the DPPH analysis, it was observed that the antioxidant activities of Alcalase hydrolysates at low concentrations were higher than those of other hydrolysates (*p* < 0.05).

On the other hand, again different from the DPPH analysis, no significant difference was observed between combined enzyme treatments (MFNAH and MFANH) at all concentrations (*p* < 0.05). The highest ABTS radical scavenging activities of MFANH and MFNAH were 99.37 ± 0.13% and 100.73 ± 0.63%, respectively, at a concentration of 2 mg/mL. The lowest IC_50_ value (the highest radical scavenging activity) of MFNH was 0.33 mg/mL among the hazelnut PI and hydrolysates, followed by 0.55, 0.55, 0.75, 0.96, 1.04, 1.37, and 1.67 mg/mL for MFNAH, MFANH, NH, AH, MFAH, MFPI, and PI, respectively.

## 4 Conclusion

In this study, hazelnut meal, a byproduct of the hazelnut oil industry, was valorized and used as a source of bioactive peptides with a special focus on their potential antiobesity and antioxidant activities. Sequential or individual hydrolysis by Neutrase and Alcalase were performed to prepare the protein hydrolysates. Microfluidization was applied to tentatively improve the hydrolysis and the functional properties of the hydrolysates. The combined Alcalase–Neutrase hydrolysis resulted with the best results regarding the DH, the inhibition of FFA release by PL, and free radical scavenging activities. The DH was positively correlated with the ESI of the hydrolysates but was negatively correlated with their EAI. Microfluidization caused unfolding of the protein structure, which resulted in the enhancement of the EAI of protein isolates and hydrolysates and the FFA release inhibition for Alcalase hydrolysates. Hazelnut protein hydrolysates from hazelnut meal may be good candidates as functional food ingredients with potential antiobesity and antioxidant properties. However, protein processing conditions (pretreatment, DH etc.) should be mastered according to the desired application of the protein hydrolysates.
